# The effect of conjugated linoleic acids on inflammation, oxidative stress, body composition and physical performance: a comprehensive review of putative molecular mechanisms

**DOI:** 10.1186/s12986-023-00758-9

**Published:** 2023-08-29

**Authors:** Husna Dharma Putera, Rumi Iqbal Doewes, Mohammed Nader Shalaby, Andrés Alexis Ramírez-Coronel, Zachary S. Clayton, Walid Kamal Abdelbasset, Saidmurodkhon S. Murtazaev, Abduladheem Turki Jalil, Pegah Rahimi, Elyas Nattagh-Eshtivani, Mahsa Malekahmadi, Naseh Pahlavani

**Affiliations:** 1 Department of Surgery, Faculty of Medicine, Lambung Mangkurat University, Banjarmasin, South Kalimantan Indonesia; 2 Faculty of Sport, Universitas Sebelas Maret, Jl. Ir. Sutami, 36A, Kentingan, Surakarta, Indonesia; 3 Biological Sciences and Sports Health Department, Faculty of Physical Education, Suez Canal University, Ismailia, Egypt; 4 Azogues Campus Nursing Career, Health and Behavior Research Group (HBR), Psychometry and Ethology Laboratory, Catholic University of Cuenca, Azogues, Ecuador; 5 Department of Integrative Physiology, University of Colorado Boulder, Boulder, CO USA; 6 Department of Health and Rehabilitation Sciences, College of Applied Medical Sciences, Prince Sattam Bin Abdulaziz University, Al Kharj, Saudi Arabia; 7 Department of Physical Therapy, Kasr Al-Aini Hospital, Cairo University, Giza, Egypt; 8 Department of Therapeutic Pediatric Dentistry, Dean of the Faculty of International Education, Tashkent State Dental Institute, Tashkent, Uzbekistan; 9 Department of Scientific Affairs, Samarkand State Medical University, Amir Temur Street 18, Samarkand, Uzbekistan; 10 Medical Laboratories Techniques Department, Al-Mustaqbal University College, Hilla, Babylon 51001 Iraq; 11 Department of Clinical Pharmacy, Faculty of Pharmacy, Kerman University of Medical Sciences, Kerman, Iran; 12 Social Development and Health Promotion Research Center, Gonabad University of Medical Sciences, Gonabad, Iran; 13 Department of Clinical Nutrition, School of Nutritional Sciences and Dietetics, Tehran University of Medical Sciences, Tehran, Iran; 14 Health Sciences Research Center, Torbat Heydariyeh University of Medical Sciences, Torbat-e Heydariyeh, Iran

**Keywords:** Conjugated linoleic acid, Inflammation, Oxidative stress, Body composition, Physical Performance

## Abstract

Conjugated linoleic acids (CLAs) are polyunsaturated fatty acids primarily found in dairy products and ruminant animal products such as beef, lamb, and butter. Supplementation of CLAs has recently become popular among athletes due to the variety of health-promoting effects, including improvements in physical performance. Preclinical and some clinical studies have shown that CLAs can reduce inflammation and oxidative stress and favorably modulate body composition and physical performance; however, the results of previously published clinical trials are mixed. Here, we performed a comprehensive review of previously published clinical trials that assessed the role of CLAs in modulating inflammation, oxidative stress, body composition, and select indices of physical performance, emphasizing the molecular mechanisms governing these changes. The findings of our review demonstrate that the effect of supplementation with CLAs on inflammation and oxidative stress is controversial, but this supplement can decrease body fat mass and increase physical performance. Future well-designed randomized clinical trials are warranted to determine the effectiveness of (1) specific doses of CLAs; (2) different dosing durations of CLAs; (3) various CLA isomers, and the exact molecular mechanisms by which CLAs positively influence oxidative stress, inflammation, body composition, and physical performance.

## Introduction

Exercise, whether recreational or competitive/professional, has become a key component of the lives of individuals worldwide. This increased participation in exercise has led to the recognition of sports as a fundamental international industry [1]. Nutrition has always been closely associated with exercise and athletics, and it is well established that diet and nutrient intake have a direct impact on athletic and physical performance [2, 3]. In recent decades, the use of dietary supplements, including minerals, vitamins, herbs or select botanicals, amino acids, and other substances, has significantly increased among athletes. This trend is largely driven by the goal of favorably modulating body composition and enhancing physical performance [4]. In general, a nutritional supplement may improve an athlete's performance by either reducing or exacerbating cellular processes that impact performance. Some of the processes that can be targeted include excess oxidative stress and chronic inflammation, as well as alterations in body composition [5–7]. For example, during strength training and moderate-to-long duration aerobic exercise, there is a substantial increase in the production of pro-inflammatory cytokines, such as interleukin-6 (IL-6) and interleukin-8 (IL-8), as well as the occurrence of oxidative stress [8]. Although the creation of inflammation and oxidative stress is necessary for physiological adaptation to exercise, if it remains chronically high, it can negatively impact exercise performance [8, 9]. Furthermore, previous studies have demonstrated that certain nutritional supplements, such as CLAs at a dosage of 1.2 mg/day for 12 weeks and creatine, can effectively decrease body fat and enhance athletic performance [10, 11]. Indeed, it may be advantageous to utilize nutritional supplements that not only help mitigate oxidative stress and inflammation but also have the potential to improve body composition and enhance physical performance. By targeting these aspects, athletes and individuals engaged in regular exercise may experience better overall training outcomes and athletic achievements. However, it's essential to approach the use of supplements with caution and under the guidance of qualified healthcare professionals to ensure safety and efficacy.

**Fig. 1:**
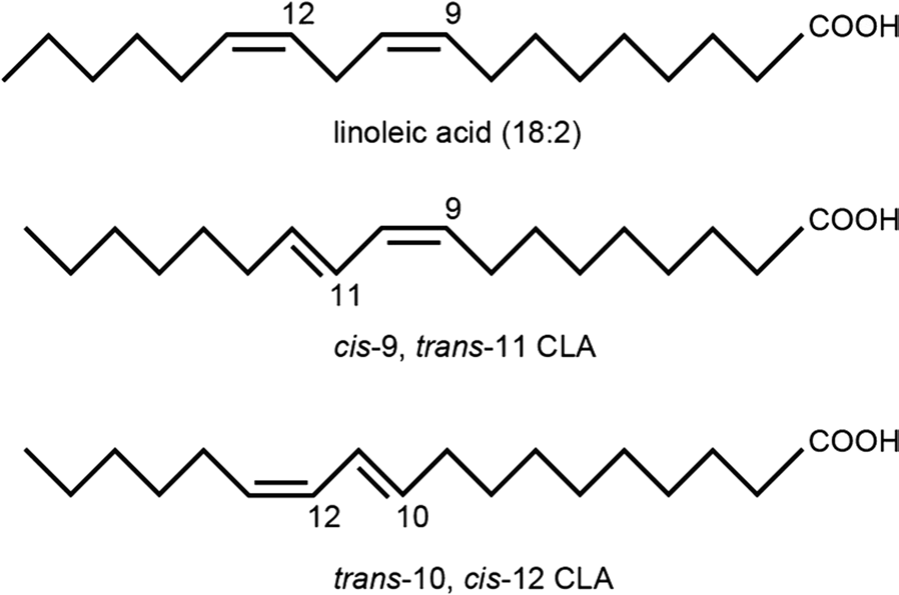
Structure of main and specific isomers of CLA

CLAs, as a nutritional supplement, have gained popularity among athletes due to their pleotropic positive effects on human health [12]. CLA is a polyunsaturated fatty acid predominantly found in dairy products and ruminant animal-based foods such as beef, lamb, and butter. Within the CLA family, there are approximately 28 different isomers [13]. The 18:2 cis-9, trans-11 (c9, t11) isomer of CLA can be obtained from the bio-hydrogenation of linoleic acid to stearic acid by ruminant bacteria. This process leads to an increase in the expression of the linoleic isomerase enzyme in ruminant bacteria, resulting in the production of the c9, t11 isomer of CLA [13]. The benefits of CLAs are thought to be elicited by its two main isomers: c9, t11-CLA and trans-10, cis-12 (t10, c12) CLA [14, 15], (Fig. 1). Indeed, there are synthetic methods for producing CLA, wherein linoleic acid-rich oils like soybean, safflower, corn, and sunflower oil are converted to CLA using an alkaline-catalyzed reaction. This process allows for the production of CLA as a dietary supplement and makes it more readily available for use in various applications [16]. According to various studies, the average daily intake of CLA is estimated to be approximately 97.5 mg/day in the UK, 35 mg/day in German women, 43 mg/day in German men, 37 mg/day in Japanese individuals, 36 mg/day in the Brazilian population, and 151–212 mg/day in Americans. These estimates indicate variations in CLA consumption across different regions and populations [17–19].

Five weeks of CLA supplementation (mixture of 39.4% c9, t11 and 38.5% t10, 12) has shown to increase lipid peroxidation, but had no effect on biomarkers of cardiovascular health, inflammation, or fasting blood glucose and insulin [20]. Indeed, the effects of CLA supplementation on body composition have shown mixed results in different studies. Some studies have demonstrated that taking CLA supplements can lead to improvements in body composition by reducing body fat percentage. However, there are also conflicting findings from other studies that have not shown consistent results in terms of body fat reduction [21–23]. Furthermore, certain studies on physical performance have shown that doses of 1.6–8.6 g/day of CLA for 3 weeks to 6 months can improve exercise outcomes, possibly due to modulated levels of testosterone, increased beta oxidation, or due to reductions in body fat percentage [22, 24, 25]. There is also evidence that CLAs can mitigate excess oxidative stress and inflammation [26–28], which may improve physical performance, but results of CLAs-mediated changes in oxidative stress and inflammation are mixed. For example, in one study supplementation with CLAs (contain 37.9% of c9, t11, and 39% of t10, c12 isomers) in mice reduced inflammation (by preventing nuclear factor kappa B (NF-κB) activation) and ultimately reduced oxidative stress [28]. Moreover, an in vitro study demonstrated that CLA (contain c9, t11 and t10, c12 isomers) in combination with linoleic acid reduces oxidative stress to a greater extent than essential fatty acids alone [29]. Also one study showed that CLA administration (50:50 isomer blend of c9, t11 and t10, c12) with 3 g/day dose after 2-months decreased inflammation and oxidative stress markers include high sensitivity C-reactive protein (hs-CRP) and malondialdehyde (MDA), and significantly increased levels of the antioxidant Glutathione peroxidase (GPx) in atherosclerosis patients, but had no effect on circulating IL-6 [26].

Although numerous studies have been conducted to assess the effects of CLAs on inflammation and oxidative stress, body composition, and physical performance, the outcomes have been inconsistent. While some studies reported positive results, others showed no significant effect. Moreover, the specific molecular mechanisms responsible for these potential effects have not been fully elucidated. Therefore, the primary objective of this review was to conduct a comprehensive examination of the existing research on the impact of CLAs on inflammation and oxidative stress, body composition, and physical performance in human studies. The focus of this review was to explore the underlying molecular mechanisms responsible for any observed effects of CLAs in these areas.

## Search strategy

Google Scholar, Scopus, PubMed, and Web of Science databases were searched to identify the relevant studies. Our keywords include “CLA,” OR “Conjugated linoleic acids,” OR “cis-9, trans-11-conjugated linoleic acid, OR “cis-9, trans-11-CLA,” OR “t10, c12—conjugated linoleic acid,” OR “t10, c12—CLA,” in combination with “Inflammation”, “Oxidative stress”, “Body composition”, “Athletic performance”, “Sport performance”, “Physical performance”, “Anthropometric indices”, “anti-oxidant”, “Inflammatory indices”, and “inflammatory markers” were used for an electronic search strategy. All the identified articles and review articles were reviewed. After that, the eligible clinical trials were selected. Also, the articles that mentioned the mechanism of the possible effect of CLA on the mentioned factors were examined. However, we declared that this study is a narrative review and not in a systematic framework.

## CLA effects on inflammation and oxidative stress

Oxidative stress can occur both chronically and acutely. It is a condition in which antioxidant enzyme defense mechanisms fail to neutralize free radicals, especially reactive oxygen species (ROS). This failure ultimately leads to an imbalance between free radicals and antioxidant defenses [30, 31]. Inflammation, which can exacerbate oxidative stress, is a complex physiological response to harmful stimuli or toxins introduced into the body [29, 32]. Moreover, inflammation can be induced by both microbial and non-microbial pathogens, tissue or cellular damage, or exposure to certain irritants [29, 32]. To complement this point, it's important to note that pro-inflammatory cytokine production can be upregulated when cells are exposed to pro-oxidant agents [33]. During prolonged and high-intensity exercise, skeletal muscles experience increased tension, leading to micro-tears in individual muscle fibers. These micro-tears subsequently trigger the release of pro-inflammatory cytokines. This cytokine release is essential for skeletal muscle recovery from exercise. However, if inflammation remains elevated chronically, it can result in a sustained state of excess inflammation and oxidative stress. Ultimately, this condition may hinder subsequent physical performance [34, 35]. Therefore, offering a solution to mitigate inflammation and oxidative stress in athletes could significantly enhance their performance, and one of these strategies involves the use of dietary supplements. One promising supplementation strategy is the utilization of select isomers of CLA, which has demonstrated anti-inflammatory and antioxidant effects [25, 36]. Antioxidant supplements have the potential to reduce oxidative stress through various mechanisms, including activating free radical scavengers, converting free radicals into inactive or less active forms, or directly binding to receptors to decrease their release [37].

The positive effects of some CLA isomer supplements on controlling inflammation and immune system responses have been demonstrated in several preclinical studies and clinical trials [17, 38–40]. Different CLA isomers have various effects on inflammation and oxidative stress, as there is evidence to suggest that isomers c9 and t11 have anti-inflammatory effects and contain approximately 80% of CLA available in natural sources, while isomers t10 and c12 are responsible for promoting weight loss [41, 42].

A study conducted in preclinical animal models demonstrated that a 50:50 isomeric blend of c9, t11, and t10, c12 CLA can reduce the production of pro-inflammatory cytokines [43], However, it should be noted that these effects have not been consistently demonstrated in all studies. For instance, Mullen et al. showed that a daily dose of 2.2 g of a 50:50 isomeric blend of c9, t11-CLA and t10, c12-CLA for 8 weeks had no significant effects on inflammatory markers when compared to a placebo [44]. In spontaneously hypertensive rats, CLA supplementation (6% sunflower oil and 1.5% of CLA as a lipid source) was found to reduce systemic inflammation, as assessed by circulating levels of tumor necrosis factor-alpha (TNF-α), in comparison to the control group (which received 7.5% sunflower oil as a lipid source) [45]. Moreover, in mice with colitis, administration of CLA (c9, t11, and t10, c12 isomers in a 50/50 ratio) at doses greater than 10 mg/day resulted in reduced inflammation and oxidative stress [46].

In patients with chronic obstructive pulmonary disease (COPD), CLA supplementation at a dose of 3.2 g/day for 6 weeks (c9, t11, and c12, t10 isomers in a 50/50 ratio) demonstrated a reduction in serum oxidative stress-related factors, including MDA and matrix metalloproteinase-9 (MMP-9), compared to the placebo group [27], The study suggested that these antioxidant effects were likely a result of the anti-inflammatory properties of CLA, which partly occur through the stimulation of peroxisome proliferator-activated receptors [27]. In another study conducted by Eftekhari et al., CLA supplementation using a 50:50 isomer blend of c9, t11, and t10, c12 at 3 g/day for 2 months resulted in decreased levels of high sensitivity C-reactive protein (hs-CRP) and MDA, while significantly increasing the levels of the antioxidant Glutathione peroxidase (GPx) in atherosclerosis patients. However, this supplementation had no effect on circulating levels of interleukin-6 (IL-6) [26]. Mohammadzadeh et al. conducted a study showing that CLA supplementation, containing isomers 18:2 c9, t11, and 18:2 t10, c12 in a 50/50 ratio, at a dose of 3 g/day for 6 weeks, was effective in reducing the levels of certain inflammatory factors in patients with rectal cancer undergoing chemotherapy. The study reported a decrease in the levels of high sensitivity C-reactive protein (hs-CRP), tumor necrosis factor-alpha (TNF-α), and matrix metalloproteinase-9 (MMP-9) [47]. Furthermore, it appears that CLA supplementation, especially with c9, t11, and t10, c12 isomers, has the potential to reduce inflammatory mediators in cancer patients. However, more studies are required to further clarify and demonstrate this effect conclusively [48]. One study involving supplementation with CLA containing c9, t11, and t10, c12 isomers in a 50:50 ratio at a dose of 6.4 g/day in obese subjects for 12 weeks showed an increase in C-reactive protein (CRP) and interleukin-6 (IL-6) levels relative to both the placebo group and the lower dose CLA group (supplemented at a dose of 3.2 g/day) [23]. Smedman et al. also showed that supplementation of CLA (containing c9,t11 and t10,c12 isomers), at a dose of 4.2 g/day after 12 weeks, increased plasma CRP but did not have a significant effect on TNF-α in healthy subjects [49]. These findings indicate that CLA (at a dose above 3.5 g/day with t10, c12 isomer) may increase levels of inflammatory and oxidative stress factors CRP and insulin resistance [50].

Furthermore, one study have shown that CLA intake lead to increase expression of glucose transporter 4 (GLUT-4) and peroxisome proliferator-activated receptor gamma (PPAR-γ) proteins in skeletal muscle during exercise [51], which is thought to be due to the role of PPAR-γ in regulating glucose homeostasis and fat metabolism, and also the interaction between PPAR-γ and GLUT-4 in insulin-mediated glucose uptake [52]. Similarly, CLA supplementation (specific isomers were not identified) during strenuous exercise has shown to reduce serum levels of high sensitivity C-reactive protein (hs-CRP), matrix metalloproteinase-2 (MMP-2), and tumor necrosis factor-alpha (TNF-α) when compared to strenuous exercise alone [53], This suggests that CLA may have anti-inflammatory and antioxidant effects during all forms of exercise. However, a previous meta-analysis indicated that supplementation with CLA (in all studies containing c9, t11 and c12, t10 isomers in a 50/50 ratio) is associated with elevated serum levels of CRP. Interestingly, it is also related to decreased serum levels of TNF-α and Interferon gamma (IFN-γ). This suggests that the effects of CLA on inflammatory markers may vary depending on the specific marker measured, and more research is needed to fully understand the mechanisms and potential implications of CLA supplementation on inflammation and immune responses [54]. In a study conducted by Song et al. CLA supplementation for 12-week (c9, t11 and c12, t10 isomers in 50/50 ratio) in healthy participants was shown to decrease circulating pro-inflammatory IL-6 levels and increase circulating levels of anti-inflammatory (IL-10), and it was concluded that the anti-inflammatory effects of CLA appear to be largely due to its c9, t11 isomer [55, 56]. In patients with allergies, the c9, t11 isomer of the CLA supplement for 12 weeks improved allergy symptoms and reduced some inflammatory factors (i.e., TNF- α and IL-5), while it increased IFN-γ levels [57]. Moreover, Joseph et al. showed that supplementation with 3.5 g/day of CLA (contain 50:50 mixture of t10, c12 and c9, t11) for 8 weeks in overweight and hyperlipidemic men had no effect on factors associated with inflammation and oxidative stress [58]. Due to the contradictory results of the effects of CLAs on inflammation and oxidative stress, the intake of this supplement to improve these cellular processes cannot be easily recommended and further studies are warranted. Table 1 shows the effects of CLA supplementation on inflammation and oxidative stress in human studies.

**Table 1:** Effects of CLA supplementation on inflammation and oxidative stress parameters in human studies

Author (year)	Country (Reference number)	Study design (sex)	Participants numbers (intervention/placebo)	Type and (dose) of CLA administered	Duration (mean age of subjects), [Health status]	Outcome measures
Turpeinen et al. (2008)	Finland [57]	RCT (M/F)	40 (20/20)	CLA capsules contained 65·3% cis-9, trans-11-CLA (2 g/d)	12-week (20–46 years) [Birch pollen allergy subjects]	IL-5 ↓ IL-6 ↔ TNF-α ↓ IFN-γ ↑
Eftekhari et al. (2013)	Iran [26]	RCT (M/F)	90 (30/30/30)	Participants were divided into 3 groups receiving 3 g/d CLA (50:50 isomer blend of cis-9 trans-11 and trans-10 cis-12) or 1 920 mg/d ω3 or placebo	2-month (54.3 years) [Atherosclerotic patients]	hs-CRP ↓ MDA ↓ GPx ↑ IL-6 ↔
Risérus et al. (2002)	Sweden [50]	RCT (M)	60 (20/20/20)	Trans 10 cis 12 isomer of CLA (3.4 g/d) or mixture isomers of CLA (3.4 g/d) or Placebo	12-week (35–65 years) [Metabolic syndrome patients]	CRP ↑ IL-6 ↑ TNF-α ↑
Ebrahimi-Mameghani et al. (2016)	Iran [59]	RCT (M/F)	38 (19/19)	Intervention group: CLA softgel 3 g/d (isomers were not identified) + weight loss diet + 400 IU vitamin E or Control group: weight loss diet + 400 IU vitamin E	8-week (20–50 years) [Non-alcoholic fatty liver disease patients]	MDA ↔ TAC ↔
Mullen et al. (2006)	Ireland [44]	RCT (M)	30 (15/15)	2.2 g/d CLA (50:50 isomeric of cis 9, trans 11 and trans trans 10, cis 12) or Placebo	8-week (40–60 years) [Healthy middle-aged males]	CRP ↔ TNF-α ↔ IL-2 ↓ IL-6 ↔ IL-10 ↔
Sluijs et al. (2010)	Netherlands [60]	RCT (M/F)	401 (201/200)	Participants received either 4 g CLA/d (2.5 g 9-cis, 11-trans CLA/d and 0.6 g 10-trans, 12-cis CLA/d) or placebo supplements	6-month (40–70 years) [Overweight and obese adults]	CRP ↔
Joseph et al. (2011)	Canada [58]	RCT (M)	27 (crossover)	Mixture of 3.5 g/d CLA (50:50) contain (cis 9, trans 11) and (trans 10, cis 12) or Placebo (safflower)	8-week (18–60 years) [Hyperlipidemic overweight men]	IL-6 ↔ hs-CRP ↔ TNF-α ↔ Ox-LDL ↔
MacRedmond et al. (2010)	Canada [61]	RCT (M/F)	26 (13/13)	4.5 g/d CLA (contain mixture of cis-9, trans-11 36.4%, and trans-10-cis-12 37.0%) or Placebo	12-week (19–40 years) [Overweight mild asthmatics patients]	IL-5 ↔ IL-6 ↔ TNF-α ↔ IFN-γ ↔ MCP-1 ↔
Steck et al. (2007)	USA [23]	RCT (M/F)	48 (16/16/16)	3.2 g/d and 6.4 g/d CLA (50:50 ratio of cis-9, trans-11 and trans-10, cis-12 isomers) or Placebo (8 g/d safflower oil)	12-week (18–50 years) [Obese individuals]	CRP ↑ IL-6 ↑
Mohammadzadeh et al. (2013)	Iran [47]	RCT (M/F)	34 (16/18)	3 g/d CLA(contained isomers 18:2 cis 9, trans 11 and 18:2 trans 10,cis 12 in a 50/50 ratio) or Placebo (sunflower oil)	6-week (60.4 years) [Rectal cancer patients]	IL-1β ↔ IL-6 ↔ TNF-α ↓ MMP-9 ↓ hs-CRP ↓ MMP-2 ↔
Smedman et al. (2005)	Sweden [49]	RCT (M/F)	53 (28/25)	4.2 g/d CLA(containing cis-9,trans-11 and trans-10,cis-12 isomers) or Placebo (olive oil)	12-week (23–63 years) [Healthy human subjects]	CRP ↑ TNF-α ↔

M, Male; F, female; RCT, randomized controlled trial; CLA, conjugated linoleic acid; IL-5, interleukin 5; MDA, malondialdehyde; FBG, fasting blood glucose; HbA1c, glycated hemoglobin A; hs-CRP, high sensitivity C-reactive protein; GPx, glutathione peroxidase; TAC, total antioxidant capacity; TNF-ɑ, tumor necrosis; Ox-LDL, Oxidised- low density lipoprotein; factor-α; IFN-γ, Interferon gamma; MCP-1, Monocyte Chemoattractant Protein-1; GLUT-4, glucose transporter 4; PPAR-γ, Peroxisome proliferator-activated receptor gamma; MMP-9, Matrix metallopeptidase 9; MMP-2, Matrix metallopeptidase 2

**Fig. 2:**
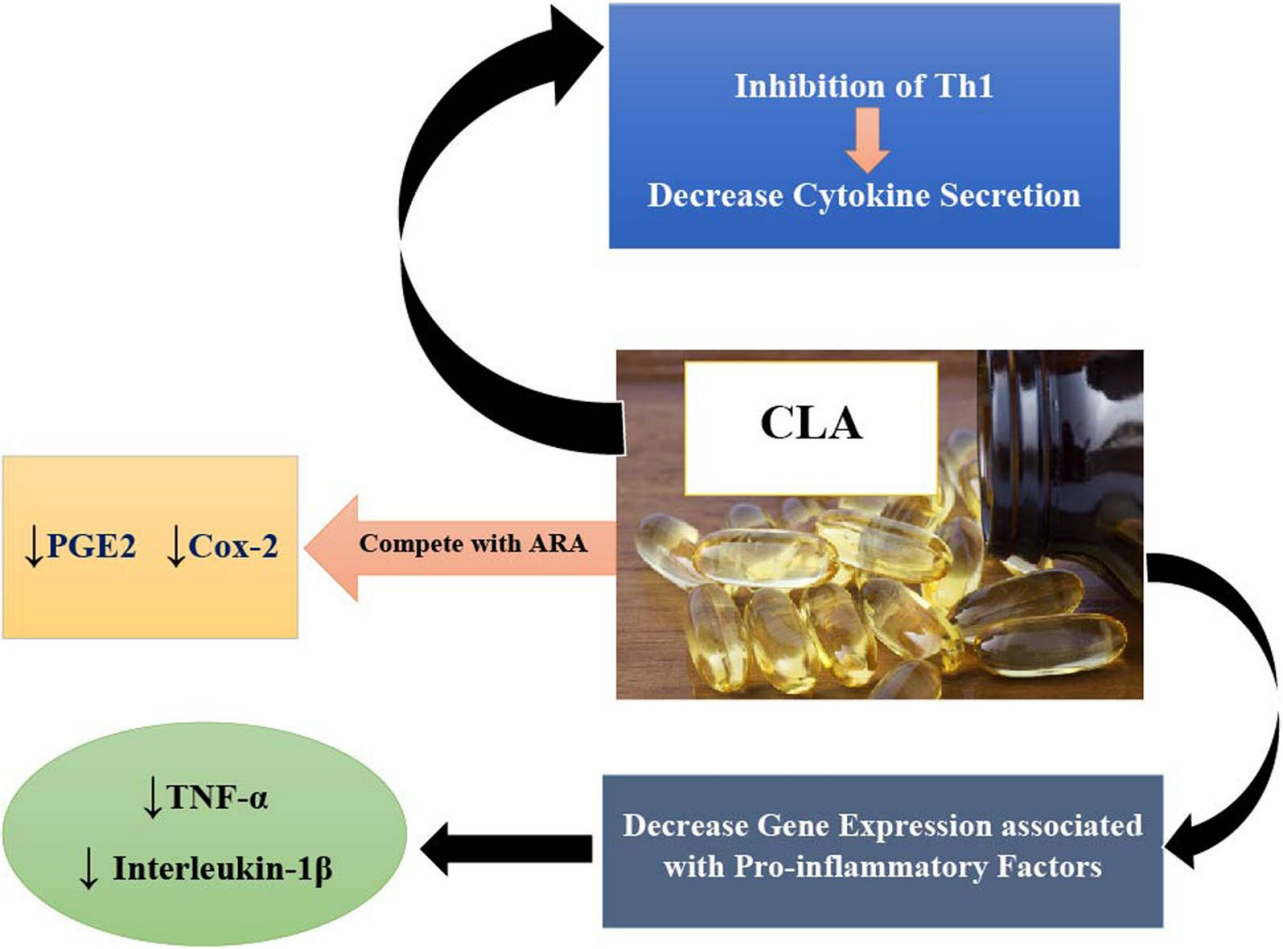
Possible molecular mechanism of the effect of CLA supplementation on inflammation and oxidative stress. ARA, Arachidonic acid; PGE2, prostaglandin E2; Cox-2, cyclooxygenase 2; Th1, type 1 T helper

There are three primary mechanisms by which CLAs are thought to reduce inflammation and oxidative stress: (1) CLA competes with linoleic acid in production of arachidonic acid, which itself is a precursor to prostaglandin E2, and thus CLA reduces the production of prostaglandins, by affecting the expression of cyclooxygenase 2 enzyme [62]; (2) Possible inhibitory effects of c9, t11 CLA isomer on type 1 T helper (Th1) cytokine secretion [63], and alteration in membrane fluidity via an effect on the concentration of essential fatty acids present in the phospholipoid plasma membrane of lymphocytes; and (3) Direct effects of CLA on the gene expression of TNF-α and IL-1β [55]. Figure 2 shows the putative mechanisms of action by which CLA may regulate inflammation and oxidative stress; however, it should be noted that all these mechanisms have been conclusively established.

## CLA effects on body composition

In previous studies, the effects of CLA on body composition, especially its effects on body fat reduction and lean body mass accrual have attracted the interest of supplement manufacturers, dietitians, and athletes [10, 64]. Animal studies have shown that t10, c12 isomer of CLA inhibits breast fat production and reduces milk fat in ruminants [65, 66], and furthermore, the administration of this isomer has been found to reduce body fat in animal studies [67–69]. Interestingly, some animal studies have shown that CLA can reduce body fat in mice, while having no effect on overall body weight [70, 71], which may suggest there was a concomitant increase in lean body mass.

In a study conducted on obese and overweight prepubertal children, CLA supplementation (t10, c12 isomer) was shown to decrease body fat (− 0.5 ± 2.1%) [72]. However, another study conducted in healthy adult women showed that CLA supplementation (50:50 c9, t11 and t10, c12 isomers) at a dose of 3 g/day for 64 days had no significant effect on body fat or fat free mass [73]. The Medstat Research and a study by Watras et al. showed that 12-weeks of CLA supplementation (39.2% c9, t11 and 38.5% t10, c12) reduced body fat by 20% in healthy men and women [74], which could be due, in part, to the duration of the intervention, type of CLA composition, differences in the type and accuracy of the tools used for body composition assessment, and the inherent participant differences (i.e., genetic backgrounds). A study by Blankson et al. showed that 3 months of CLA supplementation (contain equal parts of the c9, t11 isomer and the t10, c12 isomer) at a dose of 3.4 g/day could decrease body fat mass (− 1.30 ± 1.46 kg) [75], but there was no influence on lean body mass; however, participants did exercise throughout the supplementation period and this study did not fully identify whether these effects were related to CLA supplementation alone or due a potential synergistic effect with exercise.

MacRedmond et al. showed that 4.5 g/d of CLA (mixture of 36.4% of c9, t11, and 37.0% of t10, c12) for 12 weeks reduced body weight and BMI in asthma patients, which was associated with changes in circulating levels of the adipokines leptin and adiponectin [61]. Supplementation with CLA at a dose of 3 g/day for 4 months has shown to reduce hip circumference in obese and overweight women but have no effect on body weight, BMI, or waist circumference [76]. In another study, supplementation with CLA (isomers were not identified) for 24 months at a dose of 4.5 g/day in overweight individuals reduced body fat and lead to weight loss without any reported adverse effects [77]. Moreover, 16 weeks of CLA supplementation (8 g oil/d contain 6.4 g/d net CLA without isomers identified) in obese women with diabetes reduced BMI and total adipose mass, but had no effect on lean body mass [78], which could potentially be due to the effect of CLA on PPAR-γ, as CLA is modest ligand for PPAR-γ [79]. A meta-analysis revealed that CLA supplementation with a combination of c9, t11 and t10, c12 isomers, in a ratio of 50-to-50, can reduce overall body fat mass [80]. An additional meta-analysis showed that CLA supplementation (containing a mixture of CLA isomers particularly c9, t11 isomer in combination with t10, c12) in obese and overweight individuals significantly reduced select factors related to weight and body composition such as BMI, fat mass and body weight and also caused a gain in lean body mass, but had no influence on waist circumference [81]. It has been shown that t10, c12 CLA isomer can effect body composition by increasing fat oxidation and lipolysis [56]. One study found that the c9, t11 isomer of CLA is more involved in the anabolic process while the t10, c12 isomer is more involved in catabolic processes such as fat oxidation and lipolysis [61]. Based on the results of several human studies, supplementation with CLA or other weight loss supplements cannot reduce body weight by more than 5%, and adhering to a low-calorie diet with moderate physical activity is more effective for weight loss than supplement therapy alone [82–85]. Furthermore, it seems that the sample size, the dose and CLA isomer used, the duration of the intervention, the health status of the participants as well as the type of placebo are effective in response to treatment and are the cause of differences in the results of CLA supplementation studies. Generally, more studies are needed to determine the CLA effects on body composition changes with accurate methods and better design to evaluate and analyze these impacts in different conditions.

The putative mechanisms underlying altered body composition with CLA include reduced lipogenesis and increased lipolysis, increased expression of genes that interfere with the maturation of adipocytes [86], increased fat oxidation via elevated activity of carnitine-palmitoil-transferase-1 (CAT-1), reduced activity of lipoprotein lipase, inhibition of adipocyte differentiation, and increased activation of apoptotic pathways in adipose tissue [21, 87, 88]. Furthermore, changes in body composition may be due to changes in energy intake as a result of decreasing circulating concentrations appetite-related hormones such as leptin [73, 89]. In addition, CLA has shown to increase adiponectin, an anti-inflammatory hormone that lowers gluconeogenesis, and reduces circulating levels of leptin [77, 90]; however, one study revealed that leptin and adiponectin levels were unaffected by CLA supplementation [91]. Another plausible mechanism by which CLA modulates body composition and weight loss is related to PPARs, especially PPARγ, as CLA can reduce the expression of lipogenesis-related genes and decrease body fat by inhibiting PPARγ [92].

## CLA effects on physical performance

In recent years, supplementation with CLA and other fatty acids by athletes has received significant attention, given the influence of these supplements on favourably modulating body composition, increasing VO2max, decreasing glycogen breakdown, and ultimately improving physical performance [93, 94].

Animal studies have shown that CLA supplementation can increase testosterone secretion, which can increase energy expenditure by increasing mitochondrial biogenesis in skeletal muscle [95, 96]. CLA supplementation (isomers were not identified) in mice has shown to increase exercise capacity, improve physical performance, and promote skeletal muscle hypertrophy [95]. Moreover, CLA supplementation (only t10, c12 isomer) in mice has improved running endurance via elevated beta-oxidation in skeletal muscle-derived adipocytes and decreased hepatic glycogen breakdown [97].

A comprehensive review concluded that previous human studies have shown that administration of CLA supplements in different doses (1.6–6.8 g/day), over a period of 3–24 weeks, improves physical performance, which was associated with changes in testosterone levels [22]. Terasawa et al. showed that taking CLA supplements (isomers were not identified) at a dose of 0.9 of a gram/day for 2 weeks improved physical performance and reduced exercise-induced fatigue in male athletes [98]. Furthermore, CLA supplementation (78% total CLA, with 37% of the oil as c9, t11 isomers and 37% as t10, c12 isomers) for 6 weeks at a dose of 5.6 g/day had no effect on physical performance in young athletes, as assessed by VO2 max, physical working capacity, and gas exchange threshold [99]. In a similar study, 3 g/day of CLA supplementation (contain 22.6% t10, c12; 23.6% c11,t13; 17.6% c9, t11; 16.6% t8, c10; 7.7% t9, t11 and t10, t12; 11.9% other isomers) had no effect on physical performance and body composition in female athletes [73]. Kreider et al. and Lambert et al. demonstrated similar findings with 6.2 g/d and 3.9 g/d of CLA, and after 4 and 12-week intervention, respectively [25, 100]. Moreover, Pina et al. showed that 8 weeks of CLA supplementation at dose of 32 g/d (contain mixture of CLA isomers, predominantly the isomers c9, t11-50% and t10, c12-80%) in combination with aerobic exercise in overweight women did not have an effect on physical performance [101]. In contrary, Colakoglu et al. showed that CLA administration at a dose of 3.6 g/d (isomers were not identified) in combination with exercise (30 min daily; three times per week) for 6 weeks improved physical performance and body composition [102]. Pinkoski et al. also found that supplementation (contain all types of CLA isomers especially 36.1% c9, t11 and 36.3% t10, c12 isomers) with CLA at dose of 5 g/day for 7 weeks resulted in improved physical performance [103]. A possible reason for the CLA-mediated improvements in physical performance is the increase in fat oxidation during exercise [104].

As can be deduced from the results of various studies, CLA supplementation with doses of 1.8–6 g/day and at different times can be effective in improving endurance performance if accompanied by physical activity [10, 25, 100]. One possible explanation is that there is a synergistic effect of CLA supplementation with regular physical activity for reducing body fat and increasing lean body mass, which together could improve an athlete's performance. In general, several factors appear to mediate responsiveness to CLA supplementation, including participants' health status, dose and type of CLA supplement used, duration of intervention, level of physical activity, and age. Therefore, to show the exact effects of CLA supplementation on physical performance, future studies are needed which account for potential confounding variables. Table 2 shows the effects of CLA supplementation on body composition indices and athletic performance in human studies.

**Table 2:** Effects of CLA supplementation on body composition indices and athletic performance in human studies

Author (year)	Country (Reference number)	Study design (sex)	Participants numbers	Type and dose of CLA administered	Duration (mean age of subjects)), [Health status]	Outcome measures
Blankson et al. (2000)	Norway [75]	RCT (M/F)	47	CLA capsules (contain equal parts of the cis-9, trans-11 isomer and the trans-10, cis-12 isomer)at doses of r 1.7, 3.4, 5.1 or 6.8 g/d Placebo (9 g olive oil)	12-week (45.3 years) [Overweight and obese humans]	Body fat mass ↓ Lean body mass ↔ BMI ↔ Lipid profile ↔
Shahmirzadi et al. (2019)	Iran [105]	RCT (M/F)	54 (27/27)	CLA capsule at dose of 3 g/d containing 50:50 mixture of cis-9, trans-11 and trans-10, cis-12 CLA isomers Placebo (1500 mg/d paraffin oil)	12-week (18–45 years) [Obese Adults]	BMI ↔ Body weight ↔ Body fat mass ↓ Body fat percentage ↓ Trunk fat ↓
Sluijs et al. (2010)	Netherlands [60]	RCT (M/F)	401 (201/200)	Participants received either 4 g CLA/d (2.5 g 9-cis, 11-trans CLA/d and 0.6 g 10-trans, 12-cis CLA/d) or placebo supplements	6-month (40–70 years) [Overweight and obese adults]	Lipid profile ↔ Body composition ↔
Joseph et al. (2011)	Canada [58]	RCT (M)	27 (crossover)	Mixture of 3.5 g/d CLA (50:50) contain (cis 9, trans 11) and (trans 10, cis 12) or Placebo (safflower)	8-week (18–60 years) [Overweight, hyperlipidemic men]	Body fat mass ↔ BMI ↔ LBM ↔ Lipid profile ↔
Mądry et al. (2016)	Poland [76]	RCT (F)	62 (32/30)	3 g/d CLA (50:50 cis-9, trans-11 and trans-10, cis-12 isomers) or Placebo (sunflower oil)	12-week (54 years) [Overweight and obese women]	Body weight ↔ BMI ↔ Waist circumference ↔ Hip circumference ↓
MacRedmond et al. (2010)	Canada [61]	RCT (M/F)	26 (13/13)	4.5 g/d CLA supplementation (contain mixture of cis-9, trans-11 36.4%, and trans-10-cis-12 37.0%) or Placebo	12-week (19–40 years) [Mild asthmatics overweight subjects]	Body weight ↓ BMI ↓
Norris et al. (2009)	Germany [78]	RCT (F)	35 (crossover)	8 g/d CLA oil (net CLA;6.4 g/d), (isomers were not identified) or Placebo (Safflower oil)	16-week (59.6 years) [Obese postmenopausal type 2 diabetes women]	BMI ↓ Total adipose mass ↓ Lean body mass ↔
Colakoglu et al. (2006)	Turkey [102]	RCT (F)	44	3.6 g/d CLA(isomers were not identified) alone or in combination with exercise or Placebo	6-week (20.8 years) [Healthy female young subjects]	Body weight ↓ Fat mass ↓ WC ↓ Fat free mass ↑ Lipid profile ↔ Endurance performance ↑
Thom et al. (2001)	Norway [10]	RCT (M/F)	20 (10/10)	1.8 g/d CLA (containing equal amounts of the two isomers c9, trans 11–18:2 and trans 10, cis 12–18:2) or Placebo (hydrogel)	12-week (18–30 years) [Healthy exercising humans]	Body weight ↔ BMI ↔ Body fat percent ↓ Endurance performance ↑
Lambert et al. (2007)	South Africa [100]	RCT (M/F)	62	3.9 g/d CLA capsule (cis 9 trans 11 (29·7%) and cis 10 trans 12 (30·9%)) or Placebo (sunflower oil)	12-week (21–45 years) [Regularly exercising individuals]	Body composition ↔ Lipid profile ↔ Athletic performance ↔
Zambell et al. (2000)	USA [73]	RCT (F)	17 (10/7)	CLA capsule (contain 22.6% trans-10,cis-12; 23.6% cis-11,trans-13; 17.6% cis-9,trans-11; 16.6% trans-8,cis-10; 7.7% trans-9,trans-11 and trans10,trans-12; 11.9% other isomers), (3 g/d) or Placebo (sunflower oil)	64-day (28.3 years) [Healthy, adult women]	Body weight ↔ BMI ↔ FFM ↔ Sport performance ↔
Pina et al. (2016)	Brasil [101]	RCT (F)	28 (15/13)	3.2 g/d CLA(contain mixture of CLA isomers, predominantly the isomers cis-9, trans-11–50% and trans-10, cis-12–80%) or Placebo combining with aerobic exercise program	8-week (23 years) [Healthy overweight women]	Abdominal fat ↔ Trunk fat ↔ Athletic performance ↔
Watras et al. (2006)	USA [74]	RCT (M/F)	40 (18/22)	3.2 g/d CLA (39.2% cis-9, trans-11 and 38.5% trans-10, cis-12) or Placebo (safflower oil)	6-month (18–44 years) [Healthy, overweight subjects]	Body weight ↓ Body fat percent ↓ RMR ↔
Tajmanesh et al. (2015)	Iran [106]	RCT (M)	66 (34/32)	3.2 g day (50:50 mixture of cis-9,trans-11 and trans-10 cis-12 CLA) or Placebo (soybean oil)	8-week (20–27 years) [Healthy young men]	Maximal oxygen consumption (ml.kg.min) ↔ Time to exhaustion (min) ↔ Body weight ↔ Body mass index ↔ Waist circumference ↔
Kreider et al. (2002)	USA [25]	RCT (M)	23	CLA (6.2 g/d), (isomers were not identified) or Placebo (olive oil)	4-week (23 years) [Resistance-trained subjects]	Fat mass ↔ Body fat percent ↔ Athletic performance (strength) ↔
Jenkins et al. (2014)	USA [99]	RCT (M)	34 (18/16)	5.63 g day of total CLA isomers (of which 2.67 g was cis 9, trans 11 and 2.67 g was trans 10, cis 12) or Placebo (sunflower oil)	6-week (21.5 ± 2.8 years) [Untrained to moderately trained Healthy men]	V_O2peak (ml kg min) ↔ Respiratory compensation point (RCP) ↔ Serum cholesterol and triacylglycerol ↔
Terasawa et al. (2017)	Japan [98]	RCT (M)	10 (crossover)	Net CLA (isomers were not identified) 0.9 g/day or Placebo	2-week (Mean age not specified) [Healthy student athletes]	Body weight (increase in muscle mass) ↑ Body fat percentage ↓ Endurance performance ↑ Fatigue ↓
Pinkoski et al. (2004)	Canada [103]	RCT (M/F)	85 (43/42)	CLA at dose of 5 g/day (contain all types of CLA isomers especially 36.1% c9,t11 and 36.3% trans 10, cis 12 isomers)	7-week (18–45 years) [Resistance training subjects]	Athletic performance ↑

M, Male; F, female; RCT, randomized controlled trial; CLA, conjugated linoleic acid;; WC, waist circumference; LBM, Lean body mass; RMR, resting metabolic rate

**Fig. 3:**
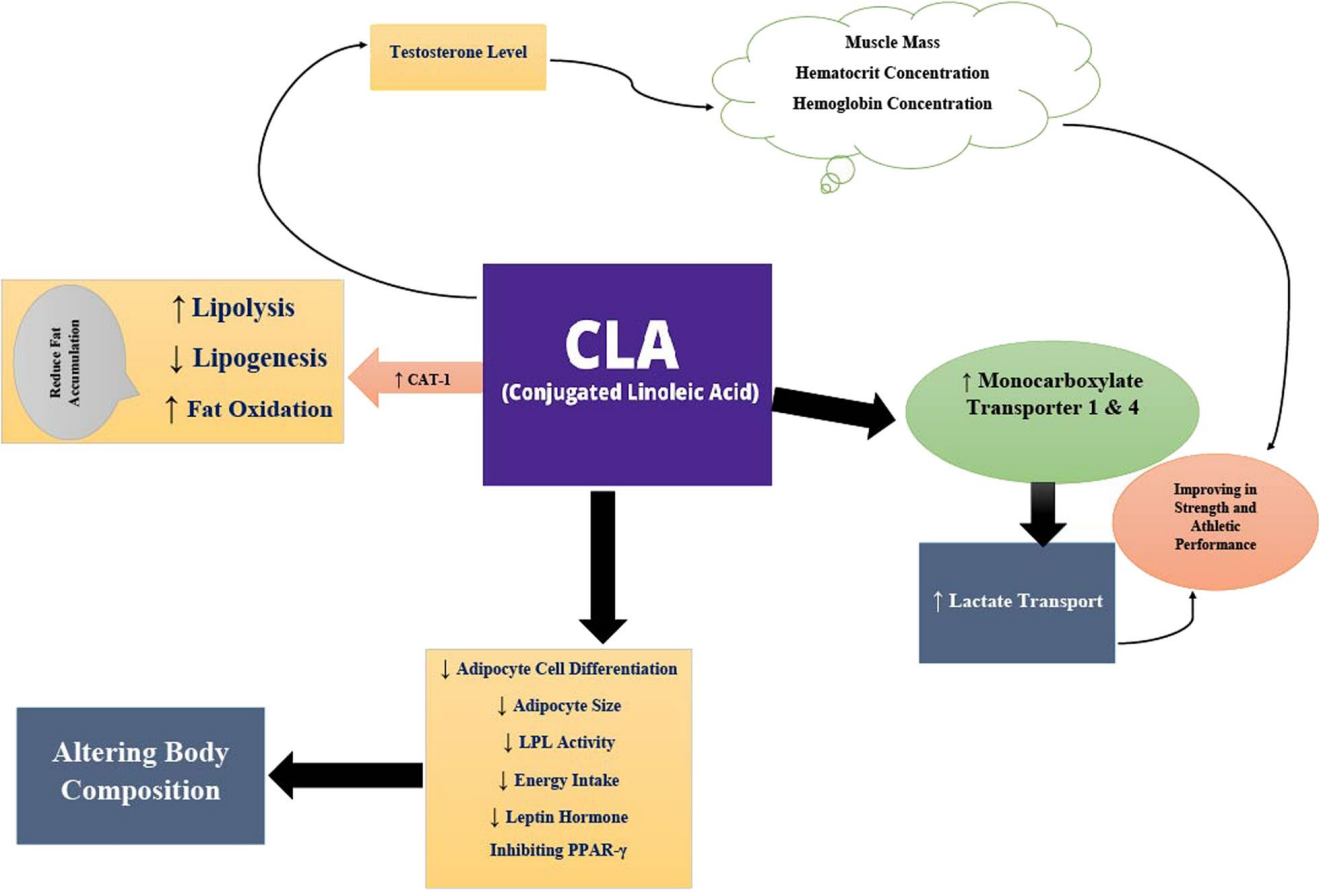
Probably mechanisms that CLA may alter the body composition and physical performance. CAT-1, Carnitine-palmitoil-transferase-1; PGE2, prostaglandin E2; Cox-2, cyclooxygenase 2; Th1, type 1 T helper; LPL, lipoprotein lipase; PPARγ, peroxisome proliferator-activated receptor γ

The primary mechanisms through which CLA is likely to have an effect on improving physical performance are a change in testosterone levels [93] (as high testosterone levels may increase muscle mass), increasing hematocrit and hemoglobin concentrations (associated with elevated erythropoietin levels), and elevating lactate transport by increasing monocarboxylate transporter 1 and 4 enzyme activity in skeletal muscle (that lead to increase exercise endurance via increasing the testosterone level) [107–110]. Currently, two mechanisms have been proposed to explain the potential link between increased testosterone and improved physical performance. Firstly, in adipocytes, perilipin and hormone-sensitive lipase (HSL) creates a protective layer on surface of lipid droplets. Under stimulation, the two proteins become hyperphosphorylated and perilipin is displaced from lipid droplets, allowing HSL to convert cholesterol esters to free cholesterol. In Leydig cells, the same pathway can stimulate testosterone production following CLA treatment. Secondly, CLA can alter steroid formation by regulating gene expression of specific enzymes and transport proteins involved in synthetic testosterone production, such as 17α-hydroxylase/17, 20 lyase (CYP17A1), which converts progesterone to androstenedione. CYP17A1 expression may directly affect testosterone [111, 112]. Figure 3 shows the possible mechanisms by which CLA supplementation may improve body composition and physical performance.

## CLA supplementation side effects

It appears that CLA supplementation is generally considered safe; however, some studies have reported adverse effects, such as gastrointestinal discomfort, diarrhea, fatigue, and nausea when CLA (a mixture of the two main isomers c9, t11, and t10, c12) is taken orally [113].

## Conclusion

Indeed, the effects of CLA supplementation on inflammation and oxidative stress remain controversial, and conclusive evidence regarding its ability to directly mitigate excess oxidative stress and inflammation is lacking. Similarly, the effects of CLA on body composition and sports performance are not entirely consistent across studies. While some research suggests that CLA may lead to a minimal increase in lean body mass and a slight decrease in BMI, fat mass, and body weight in obese and overweight subjects, the observed effects may not be substantial. It is also important to consider that the reduction in body fat mass and increase in skeletal muscle mass with CLA supplementation could contribute to improvements in physical performance. In general, the positive effects of CLA observed in preclinical animal studies tend to be more pronounced than those in human cohorts. This discrepancy may be attributed to various confounding factors in human studies, such as variations in daily physical activity, non-compliance with the exact supplement dosage, and the participants' baseline health conditions. To obtain more conclusive findings, further well-designed clinical trials are necessary. These trials should consider specific durations, isomers, and doses of CLA to better elucidate the exact effects of this supplement on inflammation, oxidative stress, body composition, and physical performance in human subjects.

## Data Availability

All data in the current review study are available from the corresponding author on reasonable request.

## Abbreviations

CLA: Conjugated linoleic acids
MDA: Malondialdehyde
FBG: Fasting blood glucose
c9, t11: Cis9, trans11
t10, c12: Trans 10, cis 12
HbA1c: Glycated hemoglobin A
hs-CRP: High sensitivity C-reactive protein
NF-κB: Nuclear factor kappa B
TAC: Total antioxidant capacity
TNF-ɑ: Tumor necrosis factor-α
IFN-γ: Interferon gamma
GLUT-4: Glucose transporter 4
PPAR-γ: Peroxisome proliferator-activated receptor gamma
WC: Waist circumference
CAT-1: Carnitine-palmitoil-transferase-1
